# Genome-Wide Identification of Histone Acetyltransferases in *Fusarium oxysporum* and Their Response to *Panax notoginseng* Notoginsenosides

**DOI:** 10.3390/jof12010071

**Published:** 2026-01-16

**Authors:** Yun-Ju Hong, Hong-Xin Liao, Jin-Rui Wen, Huan-Qi Cun, Hong-Mei Shi, Zhang-Feng Hu, Fu-Rong Xu, Sulukkana Noiprasert, Kanyaphat Apiwongsrichai, Xiao-Yun Liu, Xian Dong

**Affiliations:** 1 Yunnan Key Laboratory of Chinese Medicine Processing, Yunnan University of Chinese Medicine, Kunming 650500, China; hyj2024@126.com (Y.-J.H.);; 2 Hubei Engineering Research Center for Protection and Utilization of Special Biological Resources in the Hanjiang River Basin, College of Life Sciences, Jianghan University, Wuhan 430056, China; 3 Key Laboratory of Soybean Disease and Pest Control, Ministry of Agriculture and Rural Affairs, Nanjing Agricultural University, Nanjing 210095, China; 4 School of Integrative Medicine, Mae Fah Luang University, 333 Moo 1, Thasud, Muang, Chiang Rai 57100, Thailand

**Keywords:** histone acetyltransferases, *Fusarium oxysporum*, bioinformatics analysis, notoginsenoside, epigenetic regulation, antifungal targets

## Abstract

*Panax notoginseng*, a high-value medicinal crop, suffers substantial yield losses due to *Fusarium oxysporum*-mediated root rot, for which no molecularly defined control targets are currently available. Histone acetyltransferases (HATs) serve as crucial epigenetic regulators of fungal development and stress responses; however, their functional roles in *F. oxysporum* remain largely unexplored. In this study, we systematically identified six FoHAT genes via genome-wide analysis and classified them into evolutionarily conserved subfamilies through phylogenetic comparison with orthologs from *Saccharomyces cerevisiae*, *Homo sapiens*, and *Arabidopsis thaliana*. Structural analyses revealed distinct motif compositions and domain architectures among FoHAT members, while promoter *cis*-element profiling suggested potential subfunctionalization via stress-responsive regulatory mechanisms. Functional investigations demonstrated that major notoginsenosides present in *P. notoginseng* root exudates—R_1_, Rg_1_, Rg_2_, Re, and Rd—dynamically influenced both spore germination and FoHAT expression profiles. Intriguingly, each notoginsenoside exerted concentration-dependent non-linear effects on spore germination, either inhibiting or promoting the process. Concurrently, notoginsenoside exposure triggered compensatory transcriptional responses, most notably a rebound in *Fo-Hat1_N* expression from 9% to 112% under Rd treatment. This work establishes an initial epigenetic framework for combating Fusarium root rot in medicinal plants and offers a foundation for developing HAT-targeted small-molecule inhibitors.

## 1. Introduction

*Panax notoginseng*, a pharmacologically important member of the Araliaceae family [1], exhibits clinically validated efficacy in the management of cardiovascular and arthritic disorders [2,3]. However, its sustainable cultivation is severely hampered by *Fusarium oxysporum*-induced root rot, a disease characterized by extensive root necrosis and systemic wilting. This pathogen leads to yield reductions of 30–50% and a greater than 40% decline in medicinal notoginsenoside content, thereby undermining both therapeutic quality and economic viability [4,5]. Current management strategies face a fundamental limitation: conventional fungicides that target evolutionarily conserved pathways—such as microtubule assembly and sterol biosynthesis—not only accelerate the development of resistance but also pose significant ecotoxicological risks [6,7]. Although agroecological practices can mitigate root rot under certain conditions, their effectiveness is highly dependent on specific environmental parameters, thus limiting their utility as a mainstream control approach [5,8,9,10,11,12]. These constraints underscore the urgent need to identify pathogen-specific molecular vulnerabilities within *F. oxysporum*’s virulence apparatus—a prerequisite for developing mechanism-based antifungals that enable species-specific suppression without collateral ecological impact.

Compounding this challenge, root exudates of *P. notoginseng*—particularly amphipathic notoginsenosides (R_1_, Rg_1_, Rg_2_, Rd, and Re)—orchestrate complex rhizosphere dynamics that inadvertently increase host susceptibility to disease [13,14]. These triterpenoid compounds exhibit concentration-dependent autotoxicity, impairing root development while reshaping the soil microbiome through chemical signaling gradients [15]. Notably, notoginsenosides significantly influence the structure of soil microbial communities and regulate the composition and abundance of fungal communities via membrane interactions, thereby creating favorable conditions for pathogens. This alteration in the microbiome initiates a self-reinforcing disease cycle characterized by both a reduction in microbial diversity (Shannon index) and a decline in functional redundancy, effectively converting the rhizosphere into a niche conducive to pathogens [16]. The molecular mechanism underpinning *F. oxysporum*’s adaptation to this chemically modified environment involves its ability to epigenetically decode notoginsenoside signals—a sophisticated process mediated by histone modification-driven transcriptional plasticity that dynamically reconfigures virulence regulons. This host–pathogen signaling interface reflects an evolutionary arms race in which the pathogen’s epigenome co-opts host-derived chemistry to establish parasitic dominance, revealing a therapeutically targetable vulnerability at the epigenetic level.

Within this framework, histone acetyltransferases (HATs) act as key regulatory molecules facilitating the transduction of environmental signals into pathogenic responses. These enzymes catalyze lysine ε-amino acetylation on histone H3/H4 tails, neutralizing positive charge to relax chromatin structure and facilitate transcription of virulence genes. Phylogenetically classified into three conserved families—GNAT (regulating secondary metabolism and pathogenesis) [17,18], MYST (mediating DNA repair and developmental transitions) [19], and p300/CBP (modulating global transcription)—HATs integrate environmental cues with virulence expression [20]. Their non-redundant roles are empirically established across fungal systems: for instance, *Monascus purpureus* Δ*rtt109* mutants show altered sporulation patterns and abnormal hyphal morphology [21], while *Aspergillus fumigatus* Δ*gcnE* strains exhibit severely impaired growth, sporulation, and biofilm formation, accompanied by downregulation of key developmental regulators [18]. Despite extensive characterization in other fungi, the functional repertoire of the *F. oxysporum* HAT family (FoHATs) remains uncharacterized—a significant knowledge gap hindering the development of targeted antifungals against this economically important phytopathogen.

To address this deficit, our study integrates systematic genomic characterization of FoHATs—through phylogenetic analysis, domain architecture assessment, and *cis*-regulatory element annotation—with functional investigations using major root-exudated notoginsenosides (R_1_, Rg_1_, Rg_2_, Re, and Rd) [13,15,22]. This combined approach quantifies virulence-related phenotypes and dynamically tracks FoHAT expression patterns via RT-qPCR under notoginsenoside exposure. By establishing the first mechanistic model of pathogen–notoginsenoside crosstalk at the epigenetic interface, we provide a molecular blueprint for designing next-generation HAT-targeted inhibitors. Our work pioneers an epigenetics-based intervention strategy against Fusarium root rot, enabling precise control measures that bypass conventional resistance mechanisms and support the sustainable cultivation of this pharmacologically essential species.

## 2. Material and Methods

### 2.1. Fungal Strain Cultivation and Notoinsenoside Solution Preparation

The pathogenic fungal strain was isolated from *Panax notoginseng* roots exhibiting characteristic root rot symptoms. Genomic DNA was extracted using a published protocol [23]. The internal transcribed spacer (ITS) region was then amplified with universal primers ITS1 (5′-TCCGTAGAGAACCTGG-3′) and ITS4 (5′-TCCTCGCTTATGC-3′). Sanger sequencing of purified PCR products was conducted by Sangon Biotech (Shanghai, China). BLASTn (accessed on 19 December 2022, https://blast.ncbi.nlm.nih.gov/Blast.cgi) analysis against the National Center of Biotechnology Information (accessed on 19 December 2022, NCBI, https://www.ncbi.nlm.nih.gov/) database confirmed the isolate as *Fusarium oxysporum* (GenBank accession: OQ080022.1). Axenic cultures were maintained on potato dextrose agar (PDA) at 28 ± 1 °C.

Major root-exudate notoginsenosides were selected for functional assays: notoginsenoside R_1_ (CAS:80418-24-2, Chengdu Herbpurify (Chengdu, China), ≥98%) and notoginsenosides Rg_1_ (CAS:22427-39-0, Chengdu Must Bio (Chengdu, China), ≥98%), Rg_2_ (CAS:52286-74-5, Chengdu Must Bio, ≥98%), Re (CAS:52286-59-6, Chengdu Must Bio, ≥98%), and Rd (CAS:52705-93-8, Chengdu Must Bio, ≥98%). Individual stock solutions were prepared in chromatographic-grade methanol and stored at −20 °C. Methanol controls (CK) prepared identically served as experimental baselines.

### 2.2. Identification of HAT Genes

Candidate histone acetyltransferase genes (*FoHATs*) were systematically identified through conserved domain mining of the *F. oxysporum* FO47 genome (NCBI Taxonomy ID: 660027) using the NCBI Conserved Domain Database (accessed on 24 November 2024, https://www.ncbi.nlm.nih.gov/cdd/?term=). Search parameters targeted HAT domains across *F. oxysporum* and orthologous sequences from *Saccharomyces cerevisiae* (Taxonomy ID: 4932), *Homo sapiens* (Taxonomy ID: 9606), and *Arabidopsis thaliana* (Taxonomy ID: 3702). Following redundancy filtration and conserved domain validation, six non-redundant *FoHAT* coding sequences with unambiguous annotations were selected for further characterization.

### 2.3. Phylogenetic and Gene Family Analysis

Phylogenetic reconstruction employed protein sequence alignments of *FoHATs* and their orthologs from *Homo sapiens*, *S. cerevisiae*, and *A. thaliana* generated with MUSCLE (5.1). A neighbor-joining tree was constructed using MEGA 11 (v11.0.13) with 1000 bootstrap replicates for branch support estimation [24], with subsequent visualization and annotation performed through the TVBOT (accessed on 10 June 2024, https://www.chiplot.online/tvbot.html) online platform [25].

### 2.4. Physicochemical Characterization and Subcellular Localization

Protein physicochemical properties including molecular weight, theoretical isoelectric point, and amino acid composition were predicted via ExPASy ProtParam (accessed on 26 May 2025, https://www.expasy.org/resources/protparam) computational analysis [26]. Subcellular localization probabilities were determined using WoLF PSORT (accessed on 8 July 2025, https://www.genscript.com/wolf-psort.html) under default eukaryotic parameters [27].

### 2.5. Synteny and Gene Duplication Analysis

To investigate gene duplication events of the *FoHATs*, comparative synteny analysis was performed using the MCScanX algorithm implemented in TBtools (v2.032). Collinear blocks were identified between *F. oxysporum* and four related species—*Fusarium verticillioides* (Taxonomy ID: 117187), *Fusarium proliferatum* (Taxonomy ID: 948311), *Fusarium graminearum* (Taxonomy ID: 5518), and *Fusarium oxysporum* Fo47 (Taxonomy ID: 660027)—using genome assemblies obtained from Ensembl Fungi. The resulting syntenic relationships were visualized with the Advanced Circos module in TBtools.

### 2.6. Structural Characterization of FoHAT Genes and Conserved Domains

Gene structure analysis of *FoHATs* was performed using the GSDS 3.0 platform with *F. oxysporum* FO47 genome annotations to visualize exon–intron architectures and untranslated regions [28]. Conserved protein domains were identified through NCBI Conserved Domain Database analysis using default parameters (E-value threshold = 0.01), with subsequent structural visualizations generated in TBtools v2.032.

### 2.7. Conserved Motif Profiling and Cis-Regulatory Element Analysis of FoHATs

Evolutionarily conserved motifs were detected via MEME (accessed on 9 May 2025, https://meme-suite.org/meme/) suite analysis (v5.5.2) under standard parameters, while promoter *cis*-regulatory elements were identified from 2000 bp upstream sequences using PlantCARE (accessed on 16 January 2025, https://bioinformatics.psb.ugent.be/webtools/plantcare/html/) [28,29]. Functionally significant elements were categorized and visualized through TBtools after statistical prioritization of stress-responsive and hormone-related motifs.

### 2.8. Tertiary Structure Modeling and Protein Interaction Network Analysis of FoHATs

Protein tertiary structures were retrieved from UniProt and analyzed using PyMOL v3.0.3 for structural characterization. Protein–protein interaction networks were predicted through STRING (accessed on 7 July 2025, https://cn.string-db.org) database queries (confidence score > 0.04) and visualized using Cytoscape v19.4.1 with molecular complex detection clustering.

### 2.9. F. oxysporum Spore Response Assays to P. notoginseng Root-Exudated Notoinsenosides

For notoginsenoside response assays, *F. oxysporum* conidia were harvested from 5-day liquid cultures in potato dextrose broth (28 °C, 180 rpm) and filtered through sterile gauze. Standardized suspensions (1 × 10^6^ CFU/mL) were exposed to filter-sterilized notoginsenosides (R_1_, Rg_1_, Rd, Rg_2_, and Re) at concentrations of 0 (methanol control), 3, 6, 12, 24, and 48 μg/mL in 1/3 PDB medium. An equal amount of spore suspension was taken from the blood cell counting plate every 6–48 h, and spore germination was observed by a 10× optical microscope. Spore germination was regarded as successful when the length of the bud was more than 1/2 of the spore length. Five or more visual fields were randomly selected and the number of spore germinations was counted. The total number of spore germinations was not less than 200, and the germination kinetics were microscopically quantified, with germinated conidia defined by germ tubes exceeding half of the spore diameter. The spore germination rate was calculated by using the following formula:

D = A0/A·100%

A0: the number of germinated spores, A: the total number of spores.

### 2.10. Quantitative Real-Time Polymerase Chain Reaction Analysis of FoHAT Expression Dynamics

Transcriptional profiling employed RNA extracted using the Steadypure Plant RNA Quick Extraction Kit (Accurate Biotechnology(Hunan)co.,Ltd., Changsha, China) followed by DNase treatment. cDNA synthesis utilized Synthesis MasterMix (EG15133S, BestEnzymes Biotech Co., Ltd., Lianyungang, China). qPCR was performed on Bio-Rad CFX96 systems with ChamQ SYBR Master Mix (BestEnzymes Biotech Co., Ltd., Lianyungang, China), using *β-tubulin* (QTUB) as a reference gene. Reactions followed MIQE guidelines with three biological and technical replicates, with relative expression calculated via the 2^−ΔΔCt^ method. The primer sequences of FoHATs gene amplified by qPCR were listed in Table S1 of Supplementary Materials.

### 2.11. Quantitative Assessment and Statistical Validation

Statistical analyses were conducted using SPSS v27.0.1. Data from biological triplicates underwent ANOVA with post hoc Tukey’s tests (*p* < 0.05 significance threshold). Visualizations were generated in GraphPad Prism v9.5.1 with final composition in Adobe Illustrator v28.6.

## 3. Results

### 3.1. Phylogenetic Analysis of Fusarium oxysporum Histone Acetyltransferases

**Figure 1:**
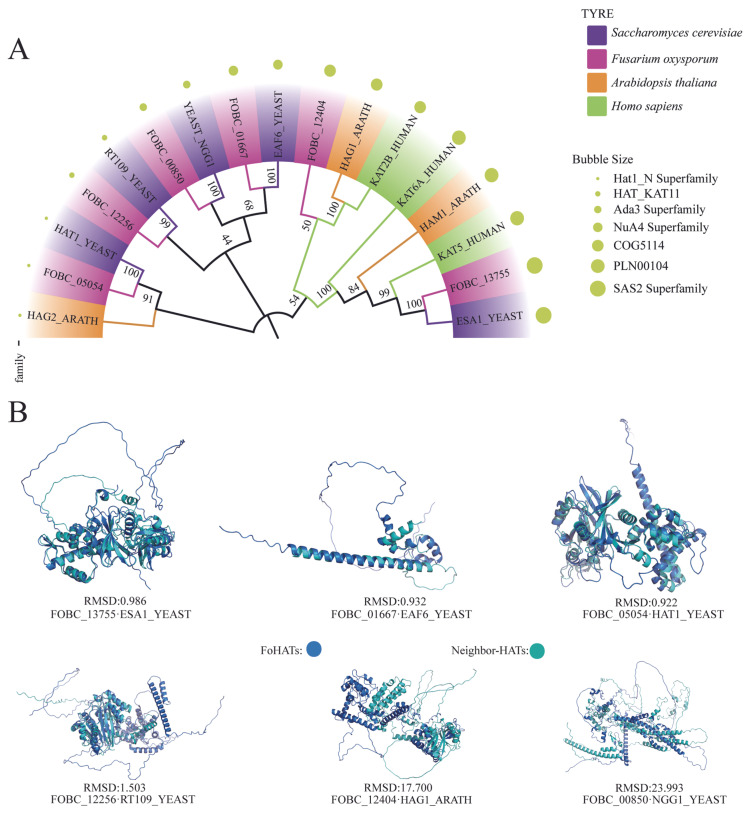
Phylogenetic and structural analysis of *Fusarium oxysporum* histone acetyltransferases (FoHATs). (**A**) Maximum-likelihood phylogeny of HAT proteins from *F. oxysporum* FO47 (bold clades), *Arabidopsis thaliana*, *Saccharomyces cerevisiae*, and *Homo sapiens*. Tree topology was constructed using the neighbor-joining method with 1000 bootstrap replicates (node values indicate support percentages). FoHATs cluster into six evolutionarily conserved superfamilies: Hat1_N, HAT_KAT11, Ada3, NuA4, COG5114, and SAS2. (**B**) Structural alignment of *FoHAT* proteins with their phylogenetic neighboring proteins was performed using PyMOL based on phylogenetic tree results. The root-mean-square deviation (RMSD) represents the average distance between superimposed atoms (typically backbone atoms) of proteins, serving as a quantitative measure of atomic positional deviations. Lower RMSD values indicate higher structural similarity between the compared proteins.

Genome-wide analysis identified six histone acetyltransferase genes (*FoHATs*) in *F. oxysporum* FO47 strain. Neighbor-joining phylogenetic reconstruction revealed evolutionary relationships among HAT orthologs across taxonomically diverse species (*Arabidopsis thaliana*, *Saccharomyces cerevisiae*, and *Homo sapiens*) (Figure 1A). The *FoHATs* clustered into six distinct conserved superfamilies, Hat1_N, HAT_KAT11, Ada3, NuA4, COG5114, and SAS2, demonstrating significant evolutionary conservation with eukaryotic reference systems. Structural validation through PyMOL-based structural alignments (using the ‘align’ command) quantified conformational homology between FoHAT proteins and their nearest phylogenetic neighbors. Root-mean-square deviation (RMSD) values ranged from 0.922 to 23.993 Å (Figure 1B), with lower values indicating higher structural conservation. Notably, FoBC_05054 exhibited exceptional structural similarity to HAT1_YEAST (RMSD = 0.922 Å), including conserved active site architecture. Conversely, the elevated RMSD (23.993 Å) between FoBC_0085 and HAT1_YEAST suggests significant structural divergence within this clade, potentially reflecting adaptive evolutionary pressures that reshaped tertiary architecture while maintaining catalytic function. This phylogenetic framework enabled standardized nomenclature for the FoHAT family (Table 1), establishing orthology relationships essential for functional characterization.

### 3.2. Biochemical Characterization and Genomic Organization of FoHATs

Comprehensive physicochemical profiling revealed significant heterogeneity within the FoHAT family (Table 1). Molecular masses ranged from 19.37 kDa (*Fo-NuA4*) to 98.95 kDa (*Fo-Ada3*), while isoelectric point analysis delineated two distinct subgroups: basic proteins (*Fo-KAT11*, pI = 9.40; *Fo-NuA4*, pI = 10.07; and *Fo-SAS2*, pI = 8.79) and acidic isoforms (*Fo-COG5114*, pI = 6.00; *Fo-Hat1_N*, pI = 5.71; and *Fo-Ada3*, pI = 5.21). Substantial length variation was observed, with *Fo-Ada3* (180 aa) and *Fo-NuA4* (637 aa) representing structural extremes.

Hydrophilicity analysis revealed negative grand average of hydropathy (GRAVY) indices for all proteins (range: −1.078 to −0.578), consistent with their hydrophilic nature and nucleoplasmic functionality. A positive grand average of hydropathy (GRAVY) value denotes hydrophobic character, whereas a negative value signifies hydrophilicity; more negative values correlate with increased hydrophilic propensity. Stability evaluation revealed that Fo-Ada3 and Fo-Hat1_N are labile proteins. In contrast, Fo-KAT11 and Fo-COG5114 exhibited high stability. According to conventional thresholds, an instability index below 40 predicts a stable protein, while a value exceeding 40 indicates potential instability [30,31].

**Figure 2:**
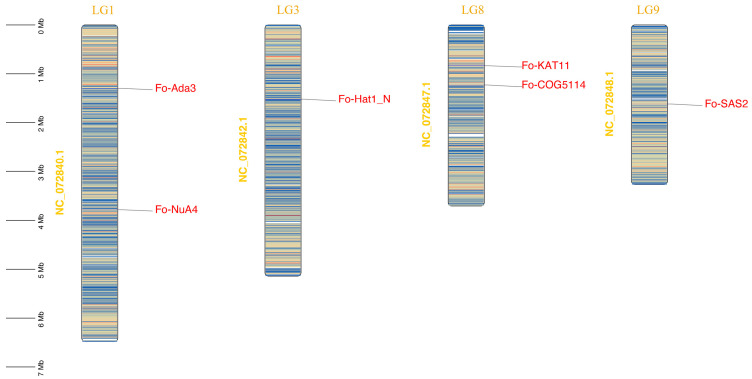
Genomic localization of *FoHAT* genes in *Fusarium oxysporum* demonstrates a non-random distribution pattern. The cytogenetic map highlights syntenic clusters of histone acetyltransferase genes. Scale bars correspond to physical distances in megabases (Mbs). Chromosomes are depicted proportionally to their actual sizes.

Aliphatic index analysis further distinguished thermotolerance potential: Fo-KAT11 (76.19), Fo-COG5114 (73.73), and Fo-Hat1_N (78.00) showed elevated indices predictive of thermal stability, contrasting with lower values in Fo-SAS2 (64.23), Fo-NuA4 (45.67), and Fo-Ada3 (53.66) [32]. Subcellular localization predictions suggested predominant nuclear localization across most *FoHATs*, with *Fo-Hat1_N* being uniquely cytoplasmic. Chromosomal mapping analysis revealed non-random genomic distribution: LG1 contained *Fo-Ada3* and *Fo-NuA4*, while LG8 harbored *Fo-KAT11* and *Fo-COG5114* spaced 0.38 Mb apart. *Fo-Hat1_N* and *Fo-SAS2* were located as singletons on LG3 and LG9, respectively (Figure 2).

### 3.3. Comparative Synteny Analysis of F. oxysporum HAT Genes

**Figure 3:**
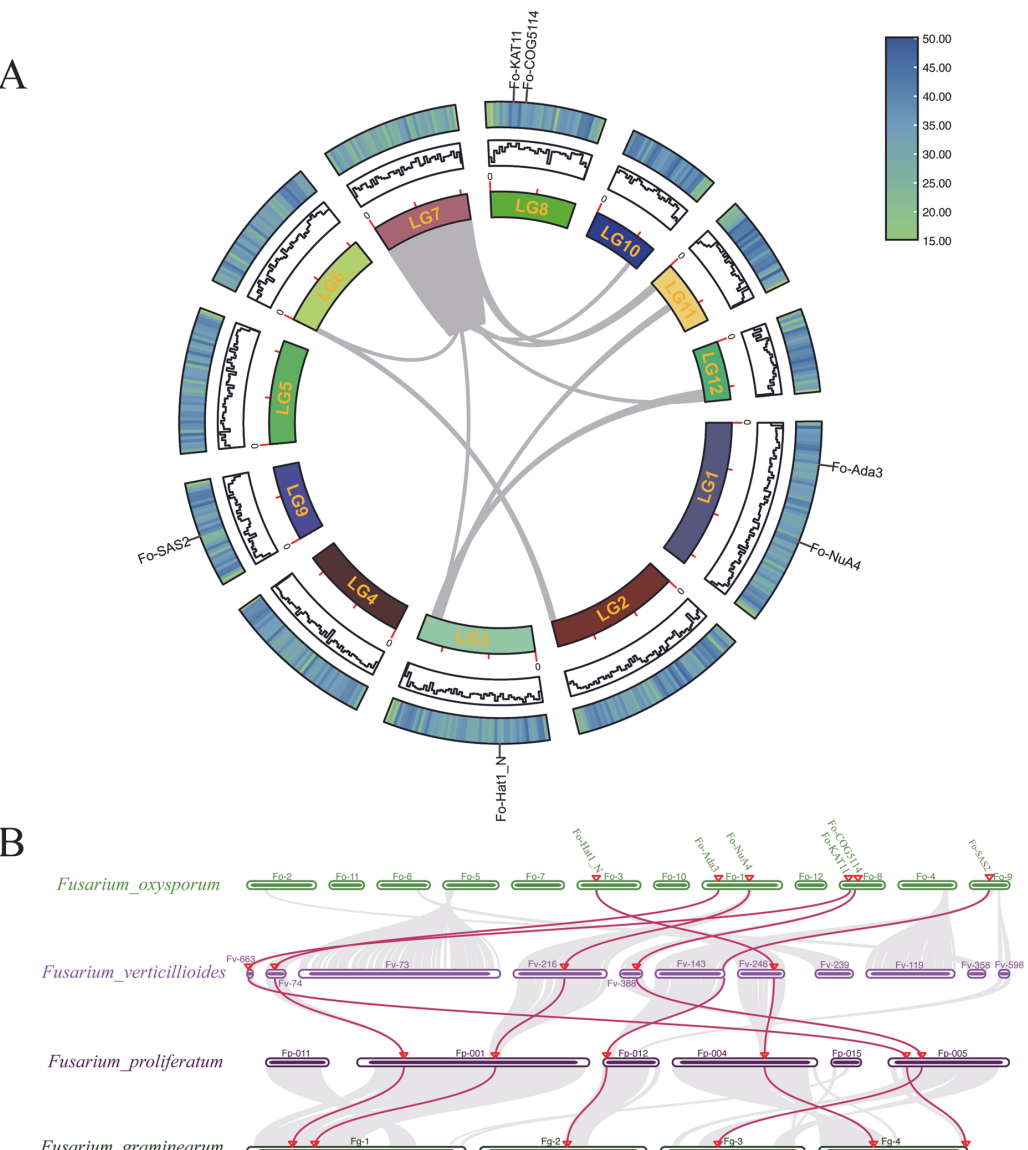
Synteny analysis of *FoHAT* genes in *Fusarium oxysporum*. (**A**) Intraspecific synteny analysis among six *FoHAT* loci. Chromosomes (LG1–LG9) are radially arranged. The absence of connecting lines indicates no significant collinear relationships. (**B**) Multispecies synteny across *Fusarium oxysporum* [Fo], *Fusarium verticillioides* [Fv], *Fusarium proliferatum* [Fp], and *Fusarium graminearum* [Fg]. Gray lines indicate background syntenic blocks. Colored lines connect putative orthologous HAT gene pairs. Red triangles mark chromosomal positions of *FoHAT* orthologs.

Genome-wide synteny assessment revealed no significant collinear relationships among the six *FoHAT* genes (Figure 3A), indicating an absence of recent duplication events within *F. oxysporum*. Phylogenetic subfamily clustering without corresponding syntenic blocks suggests divergent evolutionary trajectories rather than lineage-specific expansions.

Cross-species analysis with representative *Fusarium* taxa (*F. verticillioides*, *F. proliferatum*, and *F. graminearum*) identified six orthologous gene pairs exhibiting conserved syntenic relationships (Figure 3B). These orthologs maintained consistent chromosomal positioning across all examined species, indicating preservation from before *Fusarium* cladogenesis. The observed macro-synteny conservation supports functional maintenance of *HAT* genes during environmental adaptation and host colonization processes in these phytopathogens.

### 3.4. Structural Features, Motifs, and Conserved Domains of F. oxysporum HAT Genes

**Figure 4:**
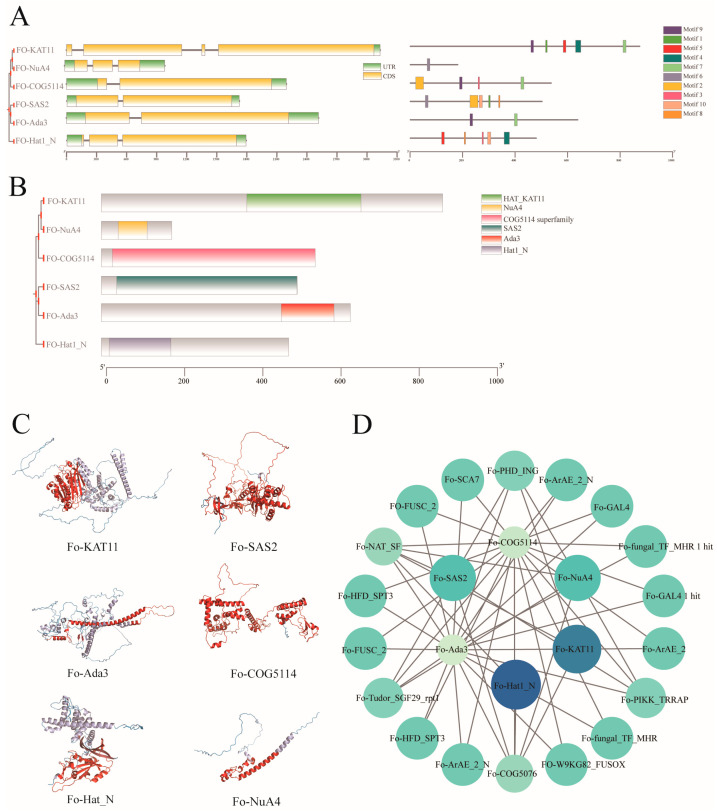
Integrated structural analysis and interaction prediction of *FoHAT* genes and proteins. (**A**) Schematic representation of gene structures for representative *FoHAT* genes (left panel). Green boxes indicate 5′ and 3′ untranslated regions (UTRs), yellow boxes represent exons, and black lines denote introns. Corresponding protein-conserved motif compositions (right panel), with Motifs 1 to 10 depicted as distinctively colored boxes. The 10 conserved motifs of *FoHATs* were analyzed in Supplementary Materials Figure S1. (**B**) Conserved protein domains identified in *FoHAT* members. Distinct colored boxes represent specific functional domains. (**C**) Predicted three-dimensional (3D) protein structures for six *FoHAT* members, with conserved structural domains highlighted in red. (**D**) Protein–protein interaction analysis of FoHAT target genes. Nodes in different colors represent *FoHAT* genes and their interacting proteins, while connecting lines indicate known or predicted interactions between corresponding proteins.

A comprehensive analysis of intron–exon architecture within the HAT gene family, guided by phylogenetic relationships (Figure 4A), revealed substantial divergence in the composition and distribution of coding sequences (CDSs) relative to untranslated regions (UTRs), despite overall structural similarities. In *Fo-KAT11*, the CDS constitutes the majority of the transcript, flanked by short UTRs at the termini. Comparable CDS proportions were observed between *Fo-SAS2* and *Fo-Ada3*, as well as between *Fo-NuA4* and *Fo-Hat1_N*. Notably, distinct exon–intron structures were evident across *FoHAT* genes, supporting the notion of functional diversification within this gene family.

MEME motif analysis identified 10 conserved motifs across HAT members (Figure 4A). The number of motifs per protein ranged from one to five, reflecting evolutionary functional specialization. *Fo-NuA4* retained only Motif 6, whereas *Fo-KAT11* (Motifs 9, 1, 5, 4, and 7), *Fo-SAS2* (Motifs 6, 2, 10, 1, and 8), and *Fo-Hat1_N* (Motifs 5, 8, 3, 10, and 4) each contained five motifs. Their complex motif architectures suggest multidomain cooperation underlying sophisticated regulatory mechanisms.

Systematic domain analysis using TBtools identified characteristic domains in *FoHATs*, including the COG5114 superfamily (*Fo-COG5114*), NuA4 (*Fo-NuA4*), SAS2 (*Fo-SAS2*), HAT-KAT11 (*Fo-KAT11*), and Ada3 (*Fo-Hat1_N*) (Figure 4B). Domain boundaries were mapped onto corresponding 3D protein structures (Figure 4C). Further examination indicated that COG5114 and SAS2 domains are distributed across multiple extended regions, suggesting roles as catalytic cores or substrate-binding modules. The *Hat1_N* domain localizes to the 5′ terminus, NuA4 and KAT11 domains cluster in central regions, and Ada3 resides toward the 3′ terminus. These domains are established key subunits of histone acetyltransferase complexes and are likely responsible for the acetylation of distinct histone targets.

Protein–Protein Interaction (PPI) networks constructed using STRING and *F. oxysporum* proteome data revealed distinct interaction profiles among HAT members (Figure 4D). *Fo-COG5114* and Fo-Ada3 functioned as central hubs, exhibiting extensive interactions with multiple proteins such as *Fo-NAT_SF*, *Fo-ARAE_2_N*, and *Fo-COG5076*. Notably, these interactions included specific binding to GAL4, the prototypic acidic activator. In contrast, *Fo-HAT1_N* interacted solely with *Fo-Nat_SF*, a member of the N-acyltransferase superfamily, and *Fo-COG5076*, a bromodomain-containing transcription factor associated with chromatin remodeling. These distinct interaction patterns indicate that *F. oxysporum* HAT members likely execute cooperative epigenetic regulation through the formation of specialized multi-protein complexes. This finding provides critical insights into the functional partitioning of HAT proteins within fungal epigenetic regulatory networks.

### 3.5. Cis-Acting Regulatory Elements in FoHAT Promoters

**Figure 5:**
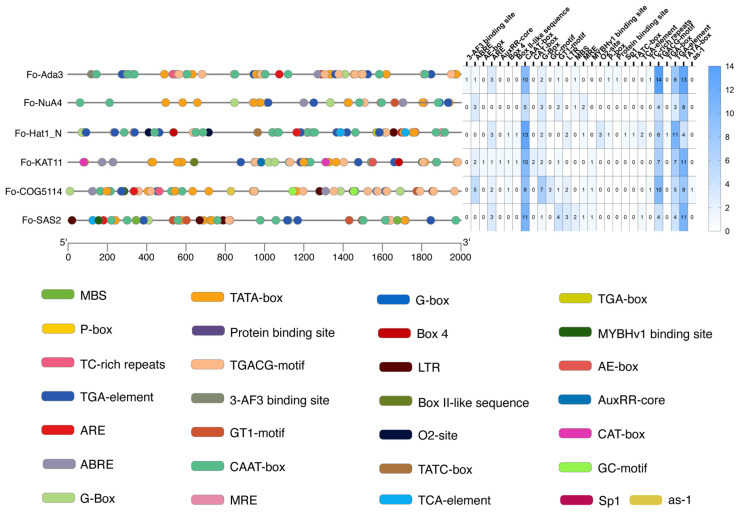
Distribution and abundance of *cis*-acting regulatory elements in *FoHAT* promoters. Schematic representation depicts approximately 2000 bp upstream genomic sequences. Horizontal lines represent promoter regions, with differently colored circular markers indicating distinct functional elements.

Analysis of promoter regions approximately 2000 bp upstream of transcription start sites using the PlantCARE database identified 29 distinct *cis*-acting regulatory elements across the *F. oxysporum* HAT family (Figure 5). Each *FoHAT* promoter contained multiple regulatory element types, indicative of complex transcriptional regulation.

All *FoHAT* genes harbored multiple TATA-box elements, for example, 13 in *Fo-Ada3* and 11 in *Fo-SAS2*, with an average density of 9.2 per promoter. The universal presence of this RNA polymerase II-binding motif indicates highly conserved basal transcriptional machinery. Similarly, CAAT-box elements were widely distributed, including 13 copies in *Fo-Hat1_N* and 11 in *Fo-SAS2*, suggesting enhanced transcriptional efficiency through NF-Y transcription factor binding. Functionally classified elements beyond these core promoters revealed three regulatory categories.

Photoregulatory elements included the G-Box with 16 total occurrences averaging 2.7 per gene. *Fo-COG5114* showed significant enrichment with seven copies while *Fo-SAS2* contained only one. Box 4 elements were detected three times collectively. The Sp1 transcription factor binding site exhibited gene-specific occurrence, detected solely in *Fo-Hat1_N* as a single copy.

Key stress-related elements comprised MYB binding sites (MBS + MYBHv1) distributed genome-wide, maximized in *Fo-SAS2* with three copies. ARE elements occurred in five genes but were absent in *Fo-NuA4*, with *Fo-Ada3* and *Fo-SAS2* each containing three copies. LTR elements showed selective distribution with two copies in *Fo-Hat1_N*, two in *Fo-COG5114*, and three in *Fo-SAS2*. Specialized elements included the as-1 exclusively in *Fo-COG5114* as a single copy, while DRE-core elements were universally absent.

Hormonal and developmental regulators included the jasmonate-responsive TGACG motif as the predominant element with 45 total occurrences averaging 7.5 per gene. *Fo-Ada3* showed marked enrichment with 14 copies. Abscisic acid-responsive ABRE elements totaled 11 occurrences, peaking in *Fo-COG5114* with 5 copies. Developmental regulation elements included CAT-box exclusively in *Fo-KAT11* with two copies. Tissue-specific O2-site elements occurred only in *Fo-Hat1_N* with three copies.

This study reveals that *FoHAT* genes maintain basal transcription through conserved TATA-box/CAAT-box architectures while achieving functional specialization via differential distribution of photoresponsive elements such as G-Box, hormone signaling components including the TGACG-motif, and stress-adaptive modules like ARE/LTR. This regulatory paradigm featuring conserved core elements alongside specialized peripheral regulators likely coordinates *F. oxysporum* adaptive responses to light perception, phytohormone signaling, and environmental stresses.

### 3.6. Modulatory Effects of Notoginsenoside on F. oxysporum Spore Germination Kinetics and Saponin Effects

**Figure 6:**
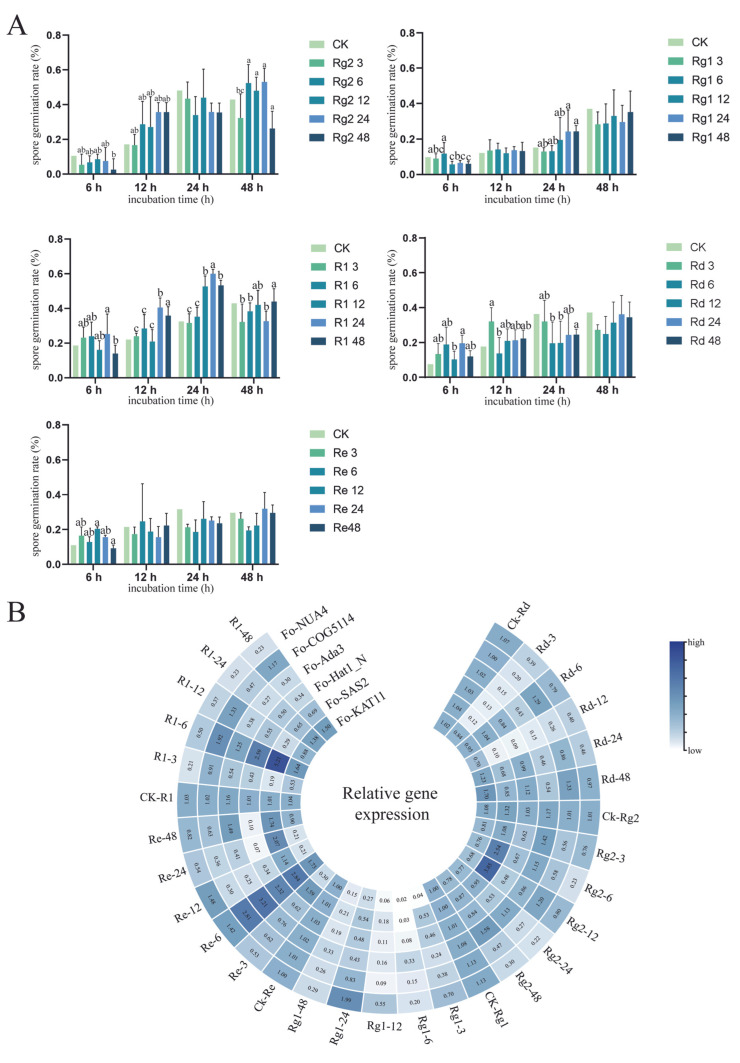
Experimental analysis of *Fusarium oxysporum* responses to *Panax notoginseng* root notoginsenoside. (**A**) Kinetics of spore germination under treatment with various notoginsenosides (R_1_, Rg_1_, Rg_2_, Re, and Rd). The *x*-axis indicates incubation time (hours), and the *y*-axis represents the spore germination rate (%). All notoginsenoside concentrations are given in μg/mL. Data points labeled with different lowercase letters denote statistically significant differences (*p* < 0.05) from the control group at the corresponding time point. (**B**) Heatmap depicting the expression profiles of *FoHAT* genes in response to different notoginsenoside types and concentrations. The *y*-axis shows the notoginsenoside treatments, and the *x*-axis lists the *FoHAT* gene identifiers. The number in the cell represents the relative expression of *FoHATs* in different treatments.

Spore germination rates increased progressively over time across all treatment groups, including the control. Treatment with notoginsenosides (Rg_1_, Rg_2_, Rd, Re, and R_1_) exhibited differential germination responses, with rates either significantly exceeding or falling below the control depending on both concentration and exposure duration (Figure 6A), indicating concentration- and time-dependent modulation of spore germination by notoginsenoside.

At 12 h, Rg_2_ treatment showed a clear dose-dependent effect: the low concentration (3 μg/mL) had no significant impact, whereas higher concentrations progressively enhanced spore germination. The 48 μg/mL R_1_ treatment transiently suppressed germination at 6 h; however, upon extension to 12 h and 24 h, germination rates surpassed those of the control, revealing a transition from inhibition to promotion. In the case of Rg_1_ at 12, 24, and 48 μg/mL, germination rates were lower than the control at 6 h. By 24 h, this inhibitory effect weakened, with germination slightly exceeding control levels, suggesting a time-dependent attenuation of inhibition. For Re treatment, a concentration-dependent regulatory effect was observed at 6 h: the low concentration (3 μg/mL) promoted germination, while the high concentration (48 μg/mL) was inhibitory. During the remaining observation periods, Re consistently exerted a mild suppressive effect on spore germination.

Transcriptional analysis revealed compound-specific patterns (Figure 6B). Under Rd treatment, *Fo-Hat1_N* expression decreased to 12.62% of control levels at 3 μg/mL but recovered to 108.73% at 48 μg/mL, suggesting a concentration-dependent induction of tolerance. Rg_2_ treatment caused potent suppression of *Fo-KAT11* expression across all concentrations (3–48 μg/mL), reaching minimum levels of 62.96% of the control without dose-dependent correlation. With the exception of the 24 μg/mL Rg_1_ treatment group, Rg_1_ consistently downregulated all *FoHAT* genes, reducing their expression to an average of 31.77% of the control. During R_1_ treatment, although spore germination shifted from inhibition to promotion across different concentration ranges, the constitutive expression of Fo-NUA4 remained consistently low, varying between 20.39% and 48.54% of control levels.

## 4. Discussion

*Panax notoginseng* is a traditional Chinese medicinal herb with high economic and practical value [1,2]. However, its cultivation is threatened by root rot caused by *Fusarium oxysporum*, and no clear control targets have been identified. Histone acetyltransferases (HATs) are key regulators of the normal physiological processes in fungi [17]. Currently, research on the biological functions of FoHATs (HATs from *F. oxysporum*) remains limited. Therefore, comprehensive bioinformatics-based identification and analysis of HAT genes in *F. oxysporum* are of great significance for developing novel control targets against *P. notoginseng* root rot and realizing the ecological cultivation of *P. notoginseng*.

The streamlined six-gene *HAT* repertoire in *F. oxysporum* (*Fo-KAT11*, *Fo-SAS2*, *Fo-COG5114*, *Fo-Hat1_N*, *Fo-Ada3*, and *Fo-NuA4*) reveals a masterclass in pathogenic genome optimization (Figure 1A). Chromosomal dispersion of these loci represents an evolutionary gambit (Figure 2)—minimizing linkage disequilibrium while liberating virulence genes for host-specific coevolution [33]. This structural parsimony extends to molecular architecture: based on comparative sequence analyses and structural characterization, elevated aliphatic indices (>70) in Fo-KAT11 and Fo-COG5114 are inferred to enhance the thermal stability of these proteins, suggesting these proteins enhance hydrophobic core stability to adapt to high-temperature environments (Table 1). This mechanism is consistent with the results that Sugarcane histone acetyltransferase 1 (*ScHAT1*) has a high aliphatic index (112.3) [34], and similarly, all 24 *GhDUF789* genes in *Gossypium hirsutum* L. display elevated aliphatic indices correlated with significant thermal stability [32].

Beyond thermodynamic optimization, subcellular localization reveals metabolic–epigenetic integration (Table 1). Cytoplasmically localized *Fo-Hat1_N* likely functions as a type B HAT, catalyzing the acetylation of free histones [35]. Similarly, *Fusarium graminearum* (Fg) possesses a homologous B-type HAT gene, *FgHAT1*, which also belongs to the HAT_N family. Targeted knockout of *FgHAT1* resulted in a Δ*FgHAT1* mutant that showed no discernible defects in growth, development, pathogenicity, or stress tolerance, indicating that *FgHAT1* is dispensable for these fundamental processes. In contrast, another B-type HAT gene, *FgHAT2*, which contains an N-terminal subunit C of the histone-binding protein RBBP4/CAF1 complex (CAF1C_H4-bd) domain and six WD40 repeats, has been demonstrated to regulate key biological functions including vegetative growth, asexual reproduction, and virulence [36]. This unique modification pattern is critical for correct histone deposition; these marks are subsequently removed [37]. This functional expansion occurs within stringent evolutionary constraints: profound synteny conservation reflects structural irreplaceability (Figure 3A). While steric precision in adenosine deaminase 2/3 (Ada2/Ada3) adaptor docking enforces molecular stasis [38], functional studies in other fungi further underscore the central role of Ada3. For instance, a histone acetyltransferase gene homologous to *ada3* has been identified in *Fusarium fujikuroi*. Subsequent functional characterization confirmed that Ada3 serves as a core subunit of the Spt-Ada-Gcn5 acetyltransferase (SAGA) complex, regulates secondary metabolite biosynthesis, and interacts with the Hat1 protein to promote transcriptional activation of the GA3 (gibberellin A3) biosynthetic gene cluster [39].

The conserved N-terminal hydrophilic cluster in *Fo-Ada3* epitomizes this evolutionary refinement. Recapitulating the catalytic core of rice *Oryza sativa* histone acetyltransferase of the GCN5 family (*OsHAG1*), this hydrophilic interface facilitates solvent-assisted allostery, with water molecules functioning as molecular lubricants during acetyl transfer [40]. Such universal preservation of acetyltransferases suggests deep evolutionary optimization where hydrogen-bonding networks fine-tune catalytic efficiency (Figure 3B). Thus, *F. oxysporum*’s epigenetic machinery emerges not through novel part invention but through strategic repurposing of conserved domains within a constrained architectural blueprint—each structural parameter fine-tuned for host niche colonization while maintaining essential functions from yeast to mammals [41].

Analysis identified ten conserved motifs within the *FoHAT* gene family, revealing an uneven distribution pattern that correlates with variations in individual gene domains. The exon count per *FoHAT* member ranges from two to four, while the intron number varies between one and three, indicating significant differences in sequence composition and genomic architecture (Figure 4A,B). Protein–protein interaction network analysis demonstrates that each *FoHAT* gene interacts with multiple functional partners. This finding closely aligns with the established assembly mechanism of histone acetyltransferase complexes (Figure 4D). Correspondingly, existing studies have shown that enzymatic activity of General control non-repressible 5 (Gcn5) is known to strictly depend on interactions with auxiliary subunits like Ada2, Ada3, and SAGA-associated factor 29 (Sgf29) [42]. Further analysis indicates that FoHAT interactors include not only direct binding partners but also numerous indirect factors, revealing a complex multi-level regulatory mechanism highly consistent with plant HAT systems. These integrated networks regulate key biological processes—including RNA metabolism, stress response, and growth and development—by converging multiple signaling pathways.

Promoter architecture reflects this functional sophistication through the integration of diverse defense signals. Promoter regions across the *FoHATs* collectively harbor 28 types of *cis*-acting elements, with each gene exhibiting a unique multi-element combinatorial profile (Figure 5). Beyond core promoter elements (TATA-box and CAAT-box), these regions are enriched with diverse response elements, such as MYB transcription factor binding sites and LTR cold-responsive elements. Their distribution patterns show striking similarity to those of plant HAT genes [40,43,44]. Notably, MYB binding sites (MBS/MYBHv1) are significantly enriched (three sites) in the *Fo-SAS2* promoter, suggesting potential involvement in MYB-mediated osmotic regulation. This observation is consistent with the molecular role of MYB transcription factors in regulating plant drought stress responses [45].

Plant defense hormones like methyl jasmonate (MeJA) and abscisic acid (ABA), originally deployed as distress signals, undergo strategic repurposing by fungal epigenomes into precise invasion coordinates—a counter intuitive transformation where host alarm systems become blueprints for pathogenic attack [46]. Such sophisticated co-option exemplifies a refined mode of epigenetic modulation in host–pathogen interactions, wherein immutable promoter cores preserve cellular homeostasis while plastic *cis*-regulatory peripheries dynamically recalibrate virulence. This operational duality provides the molecular foundation for non-linear HAT responses observed during saponin challenges, revealing how pathogens computationally hijack defensive biochemistry to steer infection trajectories. Motif combinatorics enables functional plasticity, interactome hierarchies transduce environmental cues, and promoter sequence co-option appropriates host signaling networks—collectively generating an evolutionarily adaptable epigenetic interface optimized for root colonization.

*F. oxysporum* deploys sophisticated epigenetic countermeasures against *P. notoginseng*’s saponin defenses, transforming these phytochemicals into invasion instructions through precision-tuned histone acetylation dynamics. *F. oxysporum* may balance virulence and survival by dynamically reprogramming HAT expression: at 6 h, low concentration (3 μg/mL) of Rd treatment may promote the germination process through mild metabolic stimulation, while high concentration (48 μg/mL) may trigger inhibitory checkpoints, resulting in a decrease in germination rate. Linear HAT reprogramming guides these strategic responses, leading to the inference that *FoHATs* function as critical molecular mediators of saponin chemical signal transduction. Paradoxically, *Fo-COG5114* is upregulated under R_1_ stress, which may be a compensation mechanism to maintain the basic structural and functional integrity of chromatin and ensure the transcription of essential genes. At the same time, Rg_1_ (6 μg/mL) precisely inhibited the expression of *Fo-KAT11* to a residual 2%. *Fo-Hat1_N* showed significant compensatory toughness under Rd pressure, and the expression level rebounded from 9% to 112%, revealing the alternative hyperacetylation circuit that maintains key transcription (Figure 6B). Collectively, this epigenetic regulatory system converts defensive plant specialized metabolites into spatial and temporal signals that guide invasive colonization. This exemplifies a sophisticated evolutionary adaptation through which fungal pathogens circumvent host immune responses.

Such highly variable and non-linear expression patterns suggest that notoginsenosides do not simply suppress or activate *FoHATs* universally but interfere with the histone acetylation network of *F. oxysporum* by a complex mechanism that relies on multiple genes, saponin types, and concentrations. However, given the diversity of biological processes involved in histone acetyltransferase genes, the concentration gradients (3–48 μg/mL) and time points (6, 12, 24, and 48 h) tested may not be sufficient to fully reveal the full picture of their dynamic changes. The analysis mainly focused on the transcriptional level (mRNA expression). Changes in transcription levels may not be completely equivalent to changes in protein levels and functional activity. Moreover, there was a lack of detection of epigenetic markers such as corresponding protein expression, enzyme activity, and final histone acetylation status. Future research needs to integrate proteomics and epigenetic analysis to construct a clearer regulatory network.

Given the significantly greater complexity of soil environments compared to laboratory culture conditions [47], the allelochemical interactions in the rhizosphere extend beyond notoginsenosides secreted by *P. notoginseng* roots. Other root-derived compounds, such as organic acids and triterpenoid acids, also function as important allelochemicals [48]. Furthermore, the soil system comprises a wide array of environmental participants, including root exudates from neighboring plants, diverse microbial communities, and various inherent soil compounds. The interactions among these multifaceted factors create a highly intricate soil environment.

Moreover, the combined effects of multiple microorganisms, changes in organic acid production, and fluctuations in soil pH may potentially influence the targeting efficacy of *P. notoginseng* root notoginsenoside toward HAT genes. From an ecological perspective, high concentrations of notoginsenoside may also adversely affect the normal growth of *P. notoginseng* plants, contributing to the continuous cropping obstacles commonly observed in its cultivation [49]. Therefore, the actual impacts and functions of notoginsenoside on pathogenic fungal HATs during *P. notoginseng* cultivation require further investigation and validation under corresponding environmental conditions.

## 5. Conclusions

*Fusarium oxysporum* employs a streamlined six-member HAT repertoire (*Fo-KAT11*, *Fo-SAS2*, *Fo-COG5114*, *Fo-Hat1_N*, *Fo-Ada3*, and *Fo-NuA4*) exhibiting evolutionary refinement through chromosomal dispersion and purifying selection. These epigenetic regulators integrate environmental cues via combinatorial *cis*-architectures while expanded interactomes bridge chromatin dynamics with metabolic sensing. Crucially, non-linear HAT reprogramming during saponin exposure demonstrates context-dependent plasticity: low doses prime metabolic adaptation whereas threshold concentrations (e.g., Rg_1_ at 48 μg/mL) suppress *Fo-KAT11* to 15% of the control. This work establishes fungal HATs as evolvable interfaces converting host defenses into infection strategies, nominating saponin-sensitive targets like *Fo-KAT11* for precision antifungals.

## Figures and Tables

**Table 1:** Physicochemical properties of *Fusarium oxysporum* histone acetyltransferases (FoHATs).

Gene	Gene Name	Instability Index	Protein Length P.L (aa)	Molecular Weight M.W (Da)	Isoelectric Point (PI)	Grand Average of Hydropathicity (GRAVY)	Aliphatic Index (A.I)	Localization
FOBC_12256	*Fo-KAT11*	36.40	873	98,945.19	9.40	−0.630	76.19	nuclear
FOBC_13755	*Fo-SAS2*	46.75	501	57,770.49	8.79	−0.789	64.23	nuclear
FOBC_12404	*Fo-COG5114*	40.84	536	60,333.34	6.00	−0.695	73.73	nuclear
FOBC_05054	*Fo-Hat1_N*	48.33	479	54,526.55	5.71	−0.578	78.00	cytoplasmic
FOBC_00850	*Fo-Ada3*	59.08	637	71,681.46	5.21	−1.078	53.66	nuclear
FOBC_01667	*Fo-NuA4*	42.47	180	19,370.54	10.07	−0.989	45.67	nuclear

## Data Availability

All datasets generated for this study are included in the manuscript and supplementary files. Reference genomes of *Fusarium oxysporum* FO47 (Taxonomy ID: 660027; RefSeq: GCF_013085055.1), *Saccharomyces cerevisiae* (Taxonomy ID: 4932; RefSeq: GCF_000146045.2), *Homo sapiens* (Taxonomy ID: 9606; RefSeq: GCF_000001405.40), and *Arabidopsis thaliana* (Taxonomy ID: 3702; RefSeq: GCF_000001735.4) were sourced from the NCBI database (accessed on 24 November 2024, https://www.ncbi.nlm.nih.gov/). Further inquiries can be directed to the corresponding authors.

## Supplementary Materials

The following supporting information can be downloaded at https://www.mdpi.com/article/10.3390/jof12010071/s1, Figure S1: Analysis of 10 conserved motifs in FoHATs; Table S1: Primer sequences for qPCR amplification of *FoHATs* genes; Table S2: Sequence and protein structure information.
