# Supplementary figures and images for: Genome-Wide Identification of Histone Acetyltransferases in Fusarium oxysporum and Their Response to Panax notoginseng Notoginsenosides
Source: J Fungi (Basel). 2026 Jan 16;12(1):71. doi: 10.3390/jof12010071 (PMC12843200; doi:10.3390/jof12010071)

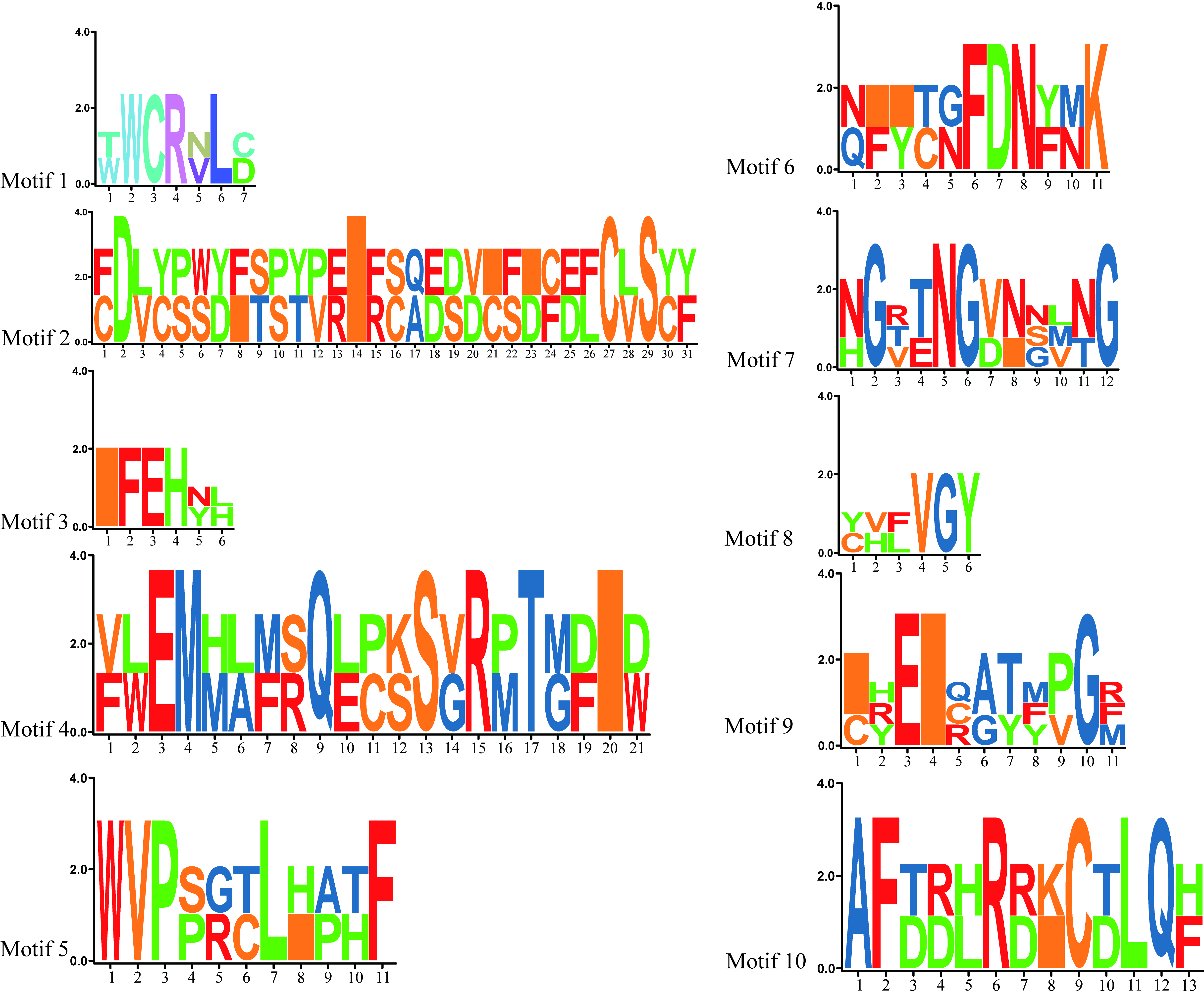

Supplement: Supplementary file 1 [file jof-12-00071-s001.zip › Figure S1.tif]
